# The effect of labor epidural analgesia on labor, delivery, and neonatal outcomes: a propensity score-matched analysis in a single Japanese institute

**DOI:** 10.1186/s40981-019-0260-z

**Published:** 2019-06-18

**Authors:** Yusuke Naito, Mitsuru Ida, Ryo Yamamoto, Kazuya Tachibana, Keiko Kinouchi

**Affiliations:** 1 Department of Anesthesiology, Osaka Women’s and Children’s Hospital, 840 Murodo-cho, Izumi-shi, Osaka Japan; 2 Department of Anesthesiology, Nara Medical University, Kashihara, Japan; 3 Department of Obstetrics, Osaka Women’s and Children’s Hospital, Izumi, Japan

**Keywords:** Epidural labor, Cesarean section, Labor, Assisted vaginal delivery, Propensity score-matched analysis

## Abstract

**Purpose:**

Lumbar epidural analgesia (LEA) is the most widely used method in reducing labor pain. Previous RCTs have shown that LEA does not increase cesarean section rates; however, the results are inconsistent and may vary depending on the different backgrounds. Therefore, we aimed to study whether LEA would affect the course of labor in our institute.

**Methods:**

Delivery records from October 2013 to April 2016 were collected. Deliveries at gestational age < 36 weeks and multiple pregnancies were excluded. All cases were divided into the non-epidural labor (NEL) group or the epidural labor (EL) group. We performed a propensity score matching analysis to balance intergroup differences. Our primary outcome was a mode of delivery (spontaneous, assisted vaginal, cesarean). Secondary outcomes were lengths of labor and outcomes of the neonates.

**Results:**

During the study period, 2632 cases met the inclusion criteria. All analyses were performed after propensity score matching (218 pairs). The percentage of assisted vaginal delivery increased by the use of LEA (11.5% in NEL group vs 25.7% in EL group; *p* < 0.001), but the rate of cesarean section was similar (12.8% vs 17.0%; *p* = 0.23). The durations of the first and second stages of labor were prolonged by the use of LEA in both primipara and multipara women. Outcomes of the neonates were similar in both groups.

**Conclusion:**

Use of LEA did not increase the rate of cesarean section when analyzed by propensity score-matched analysis in our institute.

## Introduction

Although, many methods and drugs have been developed to reduce this pain, lumbar epidural analgesia (LEA) remains the most widely used method for pain control during labor [1]. However, it remains debated whether this method is prone to causing adverse events, as local anesthetics potentially suppress the intensity of uterine contractions and decrease uterine blood flow [2]. Many randomized controlled trials (RCTs) have been performed to address this question, and a meta-analysis of the corresponding results [3] demonstrated that LEA does not adversely affect the mode of delivery, such as increasing the cesarean section rate.

A systematic review with meta-analysis is considered to provide the best evidence to address such matters, as this approach can integrate all of the relevant evidence and provide a more reliable answer than a single study. On the other hand, the result of a meta-analysis represents an average value of each study, and thus the impacts of the individual studies are diminished.

In recent times, the social background of pregnant women has changed rapidly. In Japan, the average age of pregnant women has gradually increased, reaching 30 years for nulliparas. This phenomenon is also related to the increased use of assisted reproductive technology (ART), which potentially accounts for cesarean sections due to the increase in pregnancy-induced hypertension (PIH).

Therefore, it is important to reinvestigate the effects of epidural anesthesia on labor and accumulate the related evidence. Although a high-quality RCT is desired, it is ethically difficult to randomize healthy pregnant women and may result in over-exclusion of the normal population. Propensity score matching is a statistical method for collecting data retrospectively and minimizing selective bias arising from patients’ backgrounds. Many studies have reported that propensity score matching produces results similar to those of an RCT, even though it is used in a retrospective study [4].

In the present study, we examined the effect of LEA on labor at our facility using propensity score matching.

## Materials and methods

The study was conducted at a local medical center for maternal and child health, where the average number of deliveries is 1600 per annum. The study was approved by the institutional review board of our institute (approval number: 962). Since this was an observational retrospective study that did not require patient interaction, the hospital ethics committee waived the need for obtaining written informed consent from the individual patients. The study was registered in a public clinical registration site (UMIN; registration number: 000025511) before data collection.

### Study population

A 30-month retrospective observational study was conducted on all parturients who had delivered in the labor room or in the operation room of our hospital from October 2013 to April 2016. Exclusion criteria were cases with intrauterine fetal death (IUFD), cases for artificial abortion, and cases with indications for elective cesarean delivery. Deliveries at a gestational age of less than 36 weeks and multiple pregnancies were also excluded.

### Epidural labor method

Obstetric epidural services rendered by anesthesiologists are available at our hospital 24 h per day. Initiation of LEA was considered when active labor was observed (i.e., contraction with intervals of 10 min or less). After examination by the anesthesiologist and obstetrician, LEA was performed, if appropriate. Lactate Ringer’s solution (500–1000 mL) was given prior to the initiation of LEA. After the epidural catheter had been inserted, 3 mL of 1.5% lidocaine with 18.75 μg epinephrine was administered following an aspiration test. LEA was maintained with 0.1% ropivacaine along with 2 μg/mL fentanyl. Patient-controlled analgesia (PCA) was initially given with a background infusion of 8 mL/h and a 5-mL patient-controlled bolus with a 15-min lockout time. If circulatory instability or abnormality on the cardiotocogram was observed, the infusion rate was decreased or the infusion stopped.

Midwives conducted the obstetric management of all parturients during labor, under the direct supervision of an obstetrician. Routine intrapartum management of all women included monitoring of uterine contraction and fetal heart rate with cardiotocography. The frequency and duration of uterine contractions and pelvic examination were assessed every hour, along with measurements of the vital signs and body temperature, to evaluate labor progression.

### Data collection

The records of delivery, anesthesia, and medical management of all women were reviewed for data collection. Assisted vaginal delivery was defined as the vaginal delivery assisted by the vacuum or forceps. The primary outcome of our research was the mode of delivery (spontaneous, assisted vaginal, or cesarean). We also collected data on the duration of the first and second stages of labor, the grade of perineal injury, and neonatal outcomes.

Neonatal outcomes included Apgar scores at 1 min and at 5 min, cord arterial pH, and requirement for immediate intervention, such as the use of oxygen, tracheal intubation, chest compression, and catecholamine administration. Maternal variables, such as gestational weeks, gravidity, parity, concomitant disease, age, weight, and height at the onset of labor were also collected to adjust for intergroup differences with propensity score matching.

### Statistical analysis

We used propensity score-matched analysis to balance intergroup differences. The propensity score, which represented the probability of LEA use, was estimated by multiple logistic regression analysis without regard to outcome. A fully non-parsimonious model that included all variables was developed (Table 1). These variables included maternal age, height, weight, gestational week, and maternal complications. For propensity score matching, pairs were created using the nearest neighbor method. The adequacy of propensity score-matched analysis was evaluated by the overall balance achieved in terms of a less than 10% standardized mean difference.

**Table 1:** Baseline characteristics of women between NEL and EL groups

	Unmatched population (*N* = 2632)				Propensity score-matched population (*N* = 436)			
	NEL (*N* = 2406)	EL (*N* = 226)	SMD (%)	*p* value	NEL (*N* = 218)	EL (*N* = 218)	SMD (%)	*p* value
Age	32.4 ± 5.7	34.9 ± 5.4	44.1	< 0.001	34.5 ± 5.6	34.7 ± 5.4	2.0	0.82
Height (cm)	158.2 ± 7.6	158.7 ± 8.8	6.5	0.39	158.5 ± 11.0	158.7 ± 8.9	1.7	0.86
Weight (kg)	63.0 ± 9.9	65.4 ± 11.0	24.0	0.001	64.8 ± 10.2	65.1 ± 10.7	3.7	0.71
Gestational week	39.5 ± 1.2	39.9 ± 1.2	33.3	< 0.001	39.8 ± 1.1	39.9 ± 1.2	5.8	0.55
Parity			− 12.8	0.07			− 7.3	0.30
0	894 (37.2)	102 (45.1)			92 (42.2)	99 (45.4)		
1	753 (31.3)	66 (29.2)			57 (25.2)	63 (28.9)		
2	435 (18.1)	27 (11.9)			37 (16.4)	25 (11.5)		
> 3	320 (13.3)	31 (13.7)			30 (13.3)	31 (13.7)		
Gravidity			− 32.0	< 0.001			1.4	0.22
0	1239 (51.5)	163 (72.1)			146 (67.0)	155 (71.1)		
1	818 (34.0)	41 (18.1)			54 (24.8)	41 (18.8)		
2	263(10.9)	15 (6.6)			13 (6.0)	15 (6.9)		
> 3	84 (3.5)	7 (3.1)			3 (1.4)	7 (3.2)		
Complications								
Psychiatric disorder	149 (6.2)	34 (15.0)	1.3	< 0.001	31 (14.2)	29 (13.3)	0.13	0.89
Neurological disorder	32 (1.3)	7 (3.1)	6.8	0.07	5 (2.3)	6 (2.8)	− 0.23	0.76
Hypertension	49 (2.0)	11 (4.9)	4.1	0.006				
Asthma	190 (7.9)	20 (8.8)	2.5	0.61	13 (6.0)	19 (8.7)	− 1.6	0.36
Hyperthyroidism	190 (7.9)	25 (11.1)	2.0	0.10	24 (11.0)	25 (11.5)	− 0.10	0.99
Myoma	236 (9.8)	21 (9.3)	2.5	0.81	19 (8.7)	20 (9.2)	− 0.15	0.88
PIH	112 (4.7)	14 (6.2)	3.6	0.30	14 (6.4)	14 (6.4)	0	1.00
GDM	190 (7.9)	20 (8.8)	2.5	0.61	24 (11.0)	18 (8.3)	0.80	0.33
FGR	161 (6.7)	6 (2.7)	9.7	0.02	9 (4.1)	6 (2.8)	3.6	0.60
Abortion	88 (3.7)	15 (6.6)	3.2	0.045	19 (8.7)	13 (6.0)	1.4	0.36
TPD	142 (5.9)	8 (3.5)	7.0	0.18	9 (4.1)	8 (3.7)	0.87	1.00
CHD	161 (6.7)	6 (2.7)	9.7	0.02	21 (9.6)	26 (11.9)	− 0.53	0.54
Placenta	56 (2.3)	5 (2.2)	11.3	1.00	5 (2.3)	4 (1.8)	3.6	1.00

Data are expressed as mean ± standard deviation or cases (percentage). All the variables are adjusted to achieve SMR less than 10% to reduce intergroup differences

*NEL* non-epidural labor group, *EL* epidural labor group, *SMD* standard mean difference, *PIH* pregnancy-induced hypertension, *GDM* gestational diabetes mellitus, *FRG* fetus growth reduction, *TPD* threatened preterm delivery, *CHD* congenital heart disease, *Placenta* low lying placenta or placenta previa

Data were reported as mean ± standard deviation or cases (percentage), unless indicated otherwise. Variables with normal distribution were compared with unpaired *t* tests, while non-parametric variables were compared with the Mann–Whitney *U* test. The normality of the quantitative data was not evaluated statistically to avoid multiple comparisons. Categorical data were compared with the chi-square test. Since duration of labor is a time-to-event variable, this was compared using Kaplan–Meier curves along with the log-rank test.

All statistical analyses were conducted by an investigator who was not involved in the study design, data collection, and writing of the manuscript (M.I). All statistical analyses were performed using Statistical Packages for Social Science version 23 (SPSS Inc., Chicago, IL, USA). A *p* value of < 0.05 was considered to indicate statistically significant differences.

## Results

**Fig. 1:**
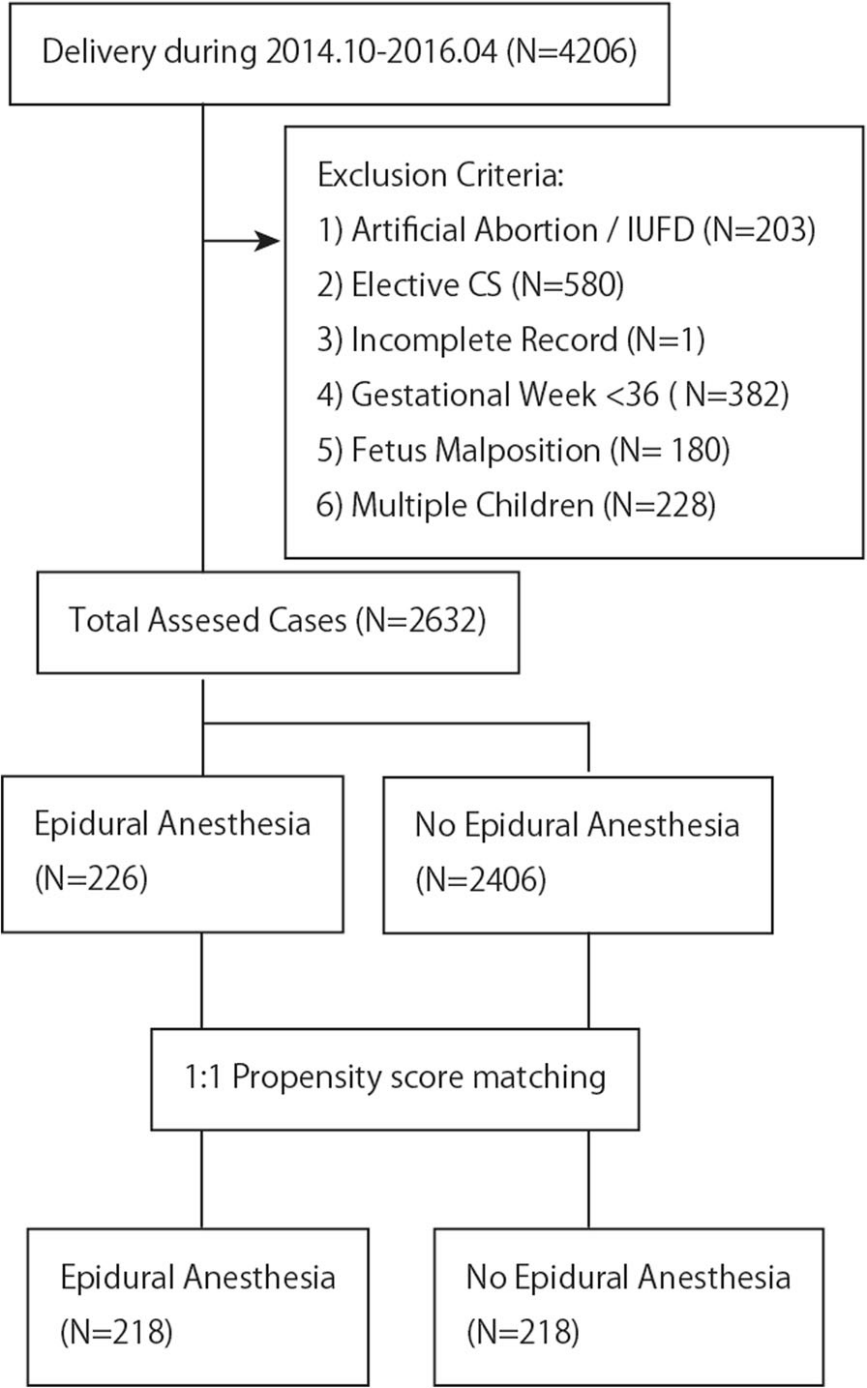
All deliveries in our institute were retrospectively investigated as shown in the flow chart. During the study period, there were 4206 deliveries. Final cases that met the criteria were 2632 cases. Within these cases, epidural analgesia was performed in 226 cases

During the study period, the total number of deliveries performed at our hospital was 4206; of these, 1574 cases met the exclusion criteria. Of the remaining 2632 cases that were included in the study population, 226 (8.6%) cases underwent epidural labor (EL group) (Fig. 1). Propensity score matching rendered 218 pairs. Before propensity score matching, there were several differences in patient characteristics between the groups (Table 1). Compared with the non-EL (NEL) group, the EL group had significantly higher maternal age; lower parity and gravidity; more frequent psychiatric complications, such as bipolar disorder, panic disorder, and schizophrenia; and fewer fetal complications. Using propensity score matching, all these differences were adjusted to small standard mean differences, suggesting that a homogeneous population had been achieved.

### Mode of delivery

The mode of delivery and complications during delivery are shown in Table 2, as the primary outcomes. Use of oxytocin was significantly associated with the use of LEA (odds ratio 0.16; 95% confidence interval 0.11–0.25). The use of LEA increased the rate of assisted vaginal delivery (odds ratio 0.37; 95% confidence interval 0.23–0.62), but not the rate of cesarean section (odds ratio 0.72; 95% confidence interval 0.42–1.22). The difference of the rates of cesarean section between groups was 4.1% (95% confidence interval − 2.6–10.8%). Grade of perineal injury was not associated with LEA use.

**Table 2:** Method of delivery and incidence of perineal injury

	NEL (*N* = 218)	EL (*N* = 218)	*p* value
Oxytocin augmentation	93 (42.7)	179 (82.1)	< 0.001
Method			
AVD	25 (11.5)	56 (25.7)	< 0.001
C/S only	28 (12.8)	37 (17.0)	0.23
Perineal injury			
None	46 (21.1)	55 (25.2)	0.27
Grade 1–2	161 (73.9)	145 (66.5)	
Grade 3–4	11 (5.0)	17 (7.8)	

The rate of cesarean delivery did not increase by the use of LEA while assisted vaginal delivery significantly increased with the use of LEA. The grades of perineal injury were similar in both groups

*SVD* spontaneous vaginal delivery, *AVD* assisted vaginal delivery, *CS* cesarean section

### Duration of the first and second stages of labor

**Fig. 2:**
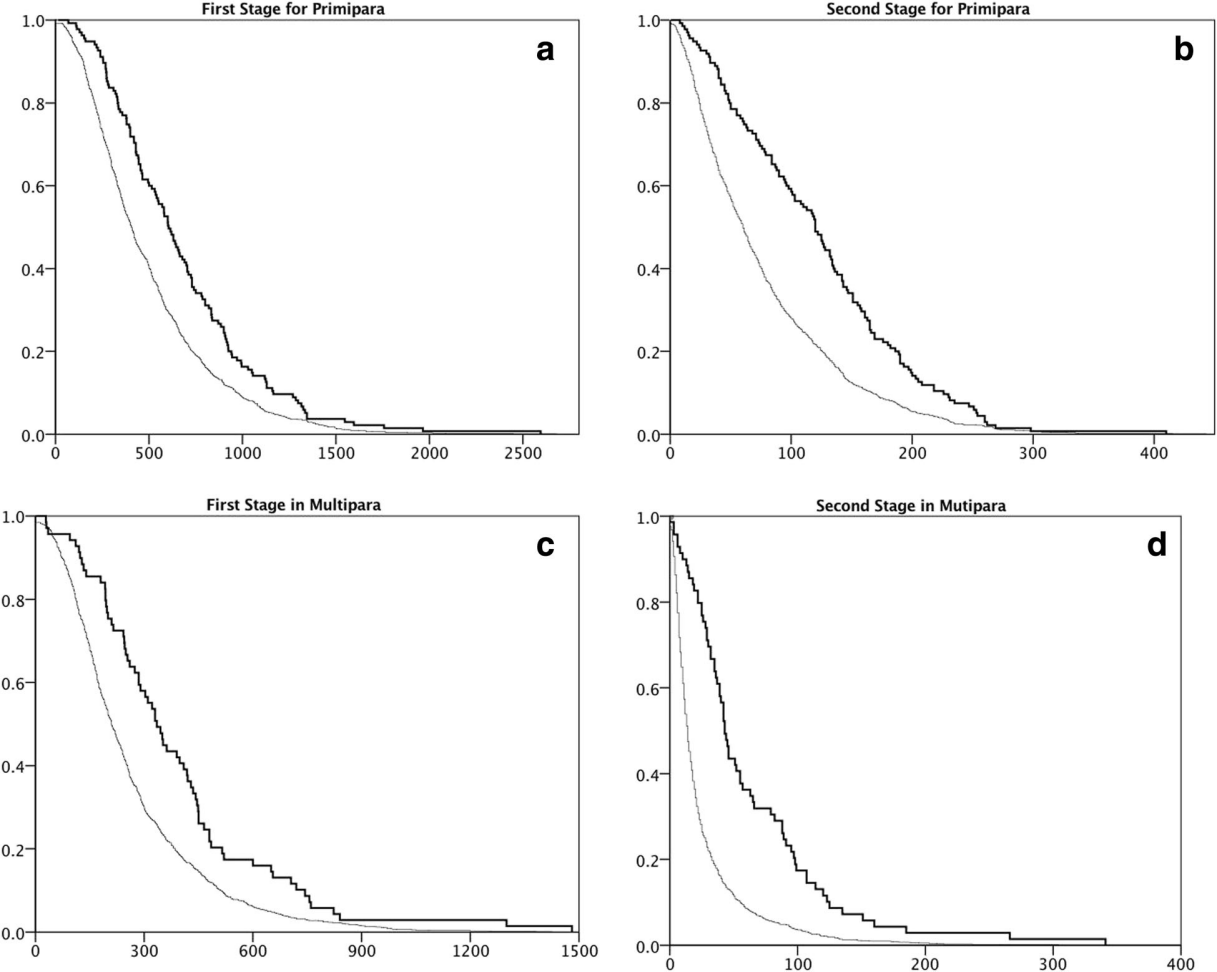
Each panel shows the Kaplan–Meier Curve for the first stage of labor for primipara (**a**), second stage for primipara (**b**), first stage for multipara (**c**), and second stage for multipara (**d**). The difference in the average time between the EL and NEL group was statistically significant between two groups compared with log-rank test (*p* < 0.01)

The duration of the first and second stages of labor was compared between the NEL and EL groups, which were subgrouped into nulliparas and multiparas (Fig. 2). The estimated length of the first stage of labor in nulliparas was 554 min (95% CI 478–630 min) in the NEL group and 972 min (95% CI 810–1134 min) in the EL group (Fig. 2a). The second stage of labor in nulliparous women lasted for 92 min (95% CI 76–108 min) in the NEL group and 177 min (95% CI 147–207 min) in the EL group (Fig. 2b). For multiparas, the first stage of labor lasted for 287 min (95% CI 230–343 min) in the NEL group and 392 min (95% CI 325–458 min) in the EL group (Fig. 2c), whereas the second stage of labor lasted for 36 min (95% CI 26–48 min in the NEL group and 70 min (95% CI 52–88 min) in the EL group (Fig. 2d). Log-rank tests showed that the differences in duration of the stages of labor between NEL group and EL group were significantly different (*p* < 0.01).

The outcomes of the neonates are shown in Table 3; there were no clinical differences between the two groups.

**Table 3:** Immediate outcome of the neonates

	NEL (*N* = 218)	EL (*N* = 218)	*p* value
Height	50.1 ± 1.9	50.3 ± 1.7	0.24
Weight	3077 ± 396	3141 ± 393	0.09
Ap < 7 (1 min)	6 (2.8)	13 (6.0)	0.08
Ap < 7 (5 min)	0 (0)	2 (0.9)	0.25
Acidocis	15 (6.9)	19 (8.7)	0.30
Intervention	42 (19.3)	54 (24.8)	0.20

There was no difference with regard to neonatal outcome between two groups. Acidosis is defined by cord arterial pH less than 7.2 immediately following delivery. Intervention refers to use of oxygen, tracheal intubation, chest compression, or use of catecholamine

## Discussion

To best of our knowledge, no previously published report had investigated the effect of epidural anesthesia on labor using propensity score matching. In this study, we failed to show an increase in the cesarean delivery rate with the use of LEA.

The results of a meta-analysis represent the integration of various studies, and thus the results should be interpreted with some caution. A specific concern with LEA research includes variations in the anesthetic drugs used, the control group, the year in which the research was performed, and in study methodology. For instance, reports from Dickinson et al. and Gambling et al. used combined spinal-epidural analgesia, rather than epidural alone [5, 6]. This could obscure the effect of pure epidural anesthesia, since spinal anesthesia is related more strongly with inhibition of motor and sympathetic nerve block, which is related to adverse events during labor. Secondly, in most studies performed in western countries, fentanyl or meperidine was used to relieve pain in the control group [7–9], while the remaining studies used no drugs in the control group [10, 11]. The difference in pain intensity among groups is problematic since the pain alters the pattern of uterine contraction. In addition, RCTs are not perfectly randomized and may include selection bias, since it is difficult to assign patients to different treatments due to ethical concerns [12].

We therefore hypothesized that a simple comparison of epidural anesthesia vs no analgesia with propensity score-matched analysis may yield different results from those previously reported, particularly in terms of cesarean delivery. However, after adjusting for patient background with propensity score matching, the incidence of cesarean section rate did not increase. These results extend those from RCTs, systematic reviews, Cochrane reviews, and impact studies.

There were some interesting features in women who chose LEA as compared with those who did not. Specifically, mothers who chose LEA were older, had less experience of delivery, and experienced more maternal complications but fewer fetal complications. In these pregnant women, the cesarean section rate was increased prior to the propensity score adjustment, but after propensity score matching, the rates of cesarean delivery between groups were comparable, and thus the cesarean section rate did not increase due to the effect of LEA itself. Moreover, the most important finding in this study is that, even when women with the above characteristics select LEA, the cesarean section rate did not rise due to the use of LEA.

In the present study, the first stage of labor was prolonged in both primiparas and multiparas. The main reason for prolonged labor is advanced maternal age. This hypothesis is partially supported by the study of Hasegawa et al., in which the average age of included women was 32, which also reported extension of the first stage of labor [13]. However, in previous studies, the impact of LEA on the first stage of labor has been controversial. Theoretically, local anesthetics prolong the first stage of labor by decreasing the intensity and frequency of uterine contraction. On the other hand, excessively strong pain during labor alters the uterine contraction pattern, which can also cause prolonged labor. Thus, it may be possible to shorten the duration of the first stage of labor by reducing pain by LEA. This suggestion is also supported by previous reports; a study with no drug administration in the control group demonstrated that the first stage of labor was shortened in the LEA group. In our study, the NEL group did not receive any type of drug to relieve pain. The reason why the duration of the first stage of labor was longer in the EL group in our study is unclear and should be further investigated in future studies.

Although the study was performed rigorously, there are some important limitations when interpreting our study. The analyses were performed after propensity score matching to reduce intergroup differences; nevertheless, our results may still be affected by confounders that we failed to collect. We were unable to collect important outcomes, such as anesthetic level during LEA and a subjective pain score. Secondly, although obstetricians conformed to the guidelines that strictly define the indications for assisted vaginal delivery and cesarean section, there are some differences among individual caregivers in clinical practice. A study by Goyert et al. has shown that, even in the same institution, the rate of cesarean section greatly varied (from 19% to 41%) between individual obstetricians, indicating that the interpretation of the guidelines may vary [14]. We chose 5% difference as clinically significant difference among groups to calculate sample size which was based on previous reports. Although our result failed to show statistical difference of cesarean section among two groups, the tendency of increased rate was rather high in the EL group. In order to clarify the effect of epidural analgesia, noninferiority clinical trials should be performed which is beyond our current study.

Finally, our result may not represent the entire population. Since few facilities perform LEA in Japan, consensus guidelines for performing LEA are lacking.

## Conclusion

The difference of the rates of cesarean section between groups was 4.1% (95% confidence interval − 2.6–10.8%). In this study, the rate of Cesarean section was not significantly different among the groups. The durations of the first and second stages of labor were prolonged by the use of LEA in both primipara and multipara women. Outcomes of the neonates were similar in both groups. Further noninferiority clinical trials are needed to confirm the effect of epidural labor.

## Data Availability

They are available as an electronic file upon reasonable request.

## Abbreviations

ART: Assisted reproductive therapy
IUFD: Intrauterine fetal death
LEA: Lumbar epidural anesthesia
PCA: Patient-controlled analgesia
PIH: Pregnancy-induced hypertension
RCT: Randomized controlled trials

## References

[1] Traynor AJ, Aragon M, Ghosh D, Choi RS, Dingmann C, Vu Tran Z (2016). Obstetric anesthesia workforce survey: a 30-year update. Anest Analg.

[2] Valentin M, Ducarme G, Ceccaldi PF, Bougeois B, Luton D (2012). Uterine artery, umbilical, and fetal cerebral Doppler velocities after epidural analgesia during labor. Int J Gynecol Obstet.

[3] Anim-Somuah M, Smyth RM, Jones L (2011). Epidural versus non-epidural or no analgesia in labour. The Cochrane database of systematic reviews.

[4] Dahabreh IJ, Sheldrick RC, Paulus JK, Chung M, Varvarigou V, Jafri H (2012). Do observational studies using propensity score methods agree with randomized trials? A systematic comparison of studies on acute coronary syndromes. Eur Heart J.

[5] Dickinson JE, Paech MJ, McDonald SJ, Evans SF (2003). Maternal satisfaction with childbirth and intrapartum analgesia in nulliparous labour. Aust N Z J Obstet Gynecol.

[6] Gambling DR, Sharma SK, Ramin SM, Lucas MJ, Leveno KJ, Wiley J (1998). A randomized study of combined spinal-epidural analgesia versus intravenous meperidine during labor: impact on cesarean delivery rate. Anesthesiology.

[7] Bofill JA, Vincent RD, Ross EL, Martin RW, Norman PF, Werhan CF (1997). Nulliparous active labor, epidural analgesia, and cesarean delivery for dystocia. Am J Obstet Gynecol.

[8] Clark A, Carr D, Loyd G, Cook V, Spinnato J (1998). The influence of epidural analgesia on cesarean delivery rates: a randomized, prospective clinical trial. Am J Obstet Gynecol.

[9] El-Kerdawy H, Farouk A (2010). Labor analgesia in preeclampsia: remifentanil patient controlled intravenous analgesia versus epidural analgesia. Middle East J Anaesthesiol.

[10] Chen LK, Hsu HW, Lin CJ, Huang CH, Tsai SK, Lee CN (2000). Effects of epidural fentanyl on labor pain during the early period of the first stage of induced labor in nulliparous women. J Formos Med Assoc.

[11] Shifman EM, Butrov AV, Floka SE, Got IB. Transient neurological symptoms in puerperas after epidural analgesia during labor. Anesteziol Reanimatol. 2007;6:17-20. 18326251

[12] Ramin SM, Gambling DR, Lucas MJ, Sharma SK, Sidawi JE, Leveno KJ (1995). Randomized trial of epidural versus intravenous analgesia during labor. Obstet Gynecols.

[13] Hasegawa J, Farina A, Turchi G, Hasegawa Y, Zanello M, Baroncini S (2013). Effects of epidural analgesia on labor length, instrumental delivery, and neonatal short-term outcome. J Anesth.

[14] Goyert GL, Bottoms SF, Treadwell MC, Nehra PC (1989). The physician factor in cesarean birth rates. N Engl J Med.

